# Special considerations in prognostic research in cancer involving genetic polymorphisms

**DOI:** 10.1186/1741-7015-11-149

**Published:** 2013-06-17

**Authors:** Sevtap Savas, Geoffrey Liu, Wei Xu

**Affiliations:** 1 Discipline of Genetics, Faculty of Medicine, Memorial University of Newfoundland, St. John’s, Newfoundland, Canada; 2 Discipline of Oncology, Faculty of Medicine, Memorial University of Newfoundland, St. John’s, Newfoundland, Canada; 3 Division of Applied Molecular Oncology, Ontario Cancer Institute, Toronto, Ontario, Canada; 4 Division of Medical Oncology and Hematology, Department of Medicine, Princess Margaret Hospital/University Health Network and University of Toronto, Toronto, Ontario, Canada; 5 Dalla Lana School of Public Health, University of Toronto, Toronto, Ontario, Canada; 6 Department of Biostatistics, Princess Margaret Hospital/University Health Network, Toronto, Ontario, Canada

**Keywords:** Genetic models, Genetic polymorphisms, Genetic prognostic factors, Genotypes, Prognostic research, Tumor DNA

## Abstract

Analysis of genetic polymorphisms may help identify putative prognostic markers and determine the biological basis of variable prognosis in patients. However, in contrast to other variables commonly used in the prognostic studies, there are special considerations when studying genetic polymorphisms. For example, variable inheritance patterns (recessive, dominant, codominant, and additive genetic models) need to be explored to identify the specific genotypes associated with the outcome. In addition, several characteristics of genetic polymorphisms, such as their minor allele frequency and linkage disequilibrium among multiple polymorphisms, and the population substructure of the cohort investigated need to be accounted for in the analyses. In addition, in cancer research due to the genomic differences between the tumor and non-tumor DNA, differences in the genetic information obtained using these tissues need to be carefully assessed in prognostic studies. In this article, we review these and other considerations specific to genetic polymorphism by focusing on genetic prognostic studies in cancer.

## Introduction

### Genetic prognostic research

Prognostic research aims to identify the factors that can predict the course of a disease in cohorts of patients [1]. Traditionally, various patient and disease-related and measurable variables, such as the demographic characteristics (for example, sex and age), pathological characteristics (for example, stage), molecular characteristics (for example, preoperative serum levels of carcinoembryonic antigen), and somatic mutations (for example, *KRAS* mutations) have been extensively investigated as prognostic markers in human cancers. While several of these variables, such as the disease stage, have been used in predicting the prognosis and outcomes in cancer patients, there is nevertheless a considerable amount of variability in clinical outcomes of patients carrying similar baseline clinicopathological characteristics. Identification of additional variables, such as genetic variations, and their inclusion into prognostic prediction models may provide better prognostic predictions and help improve treatment decisions and clinical outcomes in cancer patients [1].

In this article, we provide a review of the promises and special considerations arising from the unique features of genetic polymorphisms in prognostic research, particularly in relation to methodological and statistical applications, with an emphasis in cancer research.

### Genetic polymorphisms

The human genome contains millions of sequence and structural variations. Among the most common variations are the single nucleotide polymorphisms (SNPs: estimated number >10 million), small insertions and deletions (indels), and copy number variations (CNVs; variable number of DNA segments longer than 1 kb) [2,3]. Biological consequences of genetic and genomic variations contribute to a wide range of phenotypes, such as high-penetrant mutations observed in Mendelian diseases and low penetrant variations (also called polymorphisms) implicated in complex diseases. Therefore, many genomic variations have been extensively studied for their roles in human health and disease. In these studies, either individual alleles or genotypes at the polymorphic locus or their combinations (that is, haplotypes) at different polymorphic loci are investigated. We should mention that genetic prognostic studies benefit from the experience gained as a result of the genetic susceptibility studies investigating the genetic etiology of complex human diseases. For example, it becomes increasingly clear that in order to identify the low susceptibility alleles, more comprehensive (for example, including rare variants and structural variants, such as CNVs) and detailed (for example, investigating gene-gene and gene-environment interactions) analyses may be required [4]. In addition, since it is possible that patient prognosis can be modified by a number of different genetic variations and these risk alleles may not be shared by the individuals (that is, genetic heterogeneity), our current efforts to identify the genetic factors may be quite limited [4,5]. Genetic prognostic studies thus can learn from the strength, limitation, and experiences obtained from the genetic susceptibility studies and adapt the (emerging) concepts as relevant.

While due to the large number of variations in the human genome and their relatively poor biological characterization, the functional consequences and medical significance of a large portion of these variations are currently unknown, nevertheless, many genetic polymorphisms have been evaluated as potential prognostic markers in human diseases. In this article, for simplicity, we will use the term polymorphism to refer to any type of genetic variations that is commonly used in the contemporary research setting, regardless of their functional and phenotypic consequences. In addition, although, we will focus on SNPs, the concepts discussed in this manuscript are also applicable to other genetic variations (such as indels and CNVs).

### Univariate and multivariate analyses in prognostic research

An extensive description of the statistical tests and interpretation of their results used in prognostic studies is beyond the scope of this article. Instead, a brief, non-mathematical prologue is provided below. Interested readers may refer to other articles for further information [6-10].

Initially, association of a polymorphism and clinical outcomes is assessed through univariate analyses. Linear regression and the *t*-test are commonly used to test statistical association of continuous outcomes (for example, quality of life scores). Logistic regression and χ^2^ tests are frequently used to test statistical association of binary outcomes (for example, response rate, toxicity). Log-rank test (comparing Kaplan-Meier survival curves) and Cox regression analysis are two commonly used statistical methods to evaluate time-to-event outcomes (for example, time to progression, cancer-specific survival or overall survival). The result of these univariate analyses provides a *P* value and/or an estimated effect size (for example, odds ratio (OR) and hazard ratio (HR)) with confidence intervals that estimate whether a group of patients differs from another group of patients in terms of their prognostic characteristics. Specifically, in genetic prognostic research, these tests are used to determine whether a group of patients with a particular genotype (or genetic profiles combining multiple genotype data together) can be distinguished from patients with other genotypes or genetic profiles in terms of their outcomes.

If, after a univariate analysis, a significant association of a polymorphism with outcome is detected, then, the patients carrying a particular form of a polymorphism (for example, a homozygous or heterozygous genotype, a particular allele, or combination of alleles (for example, haplotypes)) have a poorer or better outcome than the other group of patients carrying another genotype, allele or haplotype in that cohort. However, outcome in patients are affected by many different variables (such as disease stage, age, comorbid conditions) and the compared patient groups in analyses may differ in these potentially confounding factors. Unfortunately, univariate analyses cannot adjust for these confounding factors. Thus, univariate analysis results are only the first step in analysis, helping us to understand our data and perhaps select the variables suitable for further studies, and need to be followed by multivariate analyses.

In multivariate analyses, a number of selected variables are analyzed together in a single statistical model, typically a regression model such as a logistic or Cox proportional hazard regression model. In such analyses, several variables are analyzed simultaneously to test their contribution to the outcome independent of other variables in the model. For example, the individual predictive value of a genotype may be tested after adjustment for other variables entered in the model (such as stage, age, and other clinically important variables). Genotypes that show statistically significant results after this adjustment are concluded to be independent predictors of the outcome. Another benefit of the multivariable models is that if confounding factors are entered into the multivariable analysis, then this method also helps identify the confounding factors (though in many cases some of the potential confounders remain unknown and thus cannot be included into the models).

Selection, number and characteristics of the variables entered into a multivariable model are critical in statistical analyses. For instance, to ensure a proper model, variables in the model should be kept to minimum and have relatively common variable categories (see section entitled The minor allele frequency of polymorphisms and the other determinants of the study power in the multivariable models). Several approaches are available to select the variables entered into the multivariable models, with their own advantage and disadvantage [6]. One of the ways is to enter the experimental variable (such as polymorphisms) with those variables that are shown to be independent predictors with convincing scientific evidence (such as stage in cancer). In another approach, those putative prognostic variables that have a certain *P* value in the univariate analyses (such as below 0.05 or other cut point at the discretion of the investigator) are entered into the model. If the number of variables is large, an automatic selection method (such as backward, forward, and stepwise selections) may also be performed by statistical programs to determine the variables to be entered into the final model. In another approach, all variables that cause the main association (that is, OR or HR) to change greater than 10% are included in the final model. Last but not least, all variables may be analyzed in the multivariate analysis regardless of their *P* value in the univariate analyses (that is, unselected variables). Utilization of these alternative approaches in multivariate analysis thus may result in different results.

Assuming that a multivariable model is developed, its results can pinpoint those variables that are able to predict the outcome in a study cohort independent of other prognostic variables. Once such independent predictors of outcome are identified in a study, however, validation in additional studies is required to avoid false-positive statistical results. Several approaches, such as internal and external validations, may be used in validation studies [11]. Usually, though, a model developed on a patient cohort may not work in another patient cohort with different characteristics. This is a natural anticipation as the prognosis in cancer patients is affected by many factors (such as disease and tumor characteristics, comorbid conditions, medical care, lifestyle factors, and patient ethnicity) and the distribution of such variables may differ from one cohort to other leading to different results in different cohorts. Therefore, factors that independently predict outcome regardless of this inter-patient variability are currently the ‘holy grail’ of prognostic research and whether we need a single prognostic model applicable to all patients or different models selected based on patient and disease characteristics is an ongoing debate.

In addition to validation in other patient cohorts, meta-analyses can also be useful in identifying independent prognostic markers. Incorporation of independent prognostic markers in the clinical management of patients then requires clinical utility testing, followed by consensus guidelines for clinical adaption. Although there is a tremendous amount of research performed in this field, currently the number of genetic markers used in the clinic management of patients is surprisingly small, indicating the need to design better studies and the time required to validate prognostic models in a clinically meaningful way [11-13].

### Other statistical approaches in prognostic studies

In addition to the univariate and multivariate analyses that are commonly used in prognostic studies, we should also mention other statistical approaches that are relevant to genetic prognostic studies. For example, classification and regression tree (CART) analysis is a data partitioning method that can explore the relationship between the variables and the outcome in patient cohorts [14,15]. While multivariable models concern with predicting the risk of hazard for each covariate, CART analyses are rather focused on risk stratifying (or subgrouping the patients) based on clinical and prognostic characteristics [16]. In this regard, CART analysis is useful in identifying not only the patients with similar characteristics but also the predictive capacity of covariates. Another advantage of the CART analysis is its ability to detect the interactions between the covariates included in the analysis. For example, interactions between genetic polymorphisms or between genetic polymorphisms and environmental factors may be explored using this method [17,18]. Thus, CART analysis can be useful in genetic prognostic studies as well and is suggested to be complementary to the multivariable analyses [16,19].

### Special considerations and analyses in genetic prognostic research involving genetic polymorphisms in cancer

In many ways, genetic polymorphisms differ from other potential or established prognostic markers used in prognostic studies. While some of these characteristics ease our research and thus are advantageous, others still are challenging and may need to be debated within the scientific community to develop or establish ways to address them. In the following session, we discuss these characteristics and summarize the current challenges and solutions to some of these issues (Table 1).

**Table 1:** A summary of special considerations in genetic prognostic studies

		
**Characteristics/challenge**	**Possible solutions**	**Potential benefits of the solution in genetic prognostic studies**
Correlation among genetic polymorphisms	(i) Utilization of the linkage disequilibrium (LD) information and investigating the tagging single nucleotide polymorphisms (tagSNPs) instead can prevent this issue [23]	(i) reduces the redundancy among variables and simplify the analysis while also reducing the genotyping cost and efforts [23]
	(ii) Once an association is found with a genetic polymorphism, this genomic region (usually within the same LD block) may be investigated in detail to identify the nearby ‘true’ prognostic factor that modifies the prognosis in patients	(ii) may identify the prognostic factor biologically linked to variable prognosis in patients
Genetic polymorphisms as confounders	Some of the genetic polymorphisms confounding the relationship between the prognostic factor and the outcome are likely to be in close vicinity and can be identified by investigating the genomic region in detail	Genetic confounders can be identified
Hardy-Weinberg equilibrium (HWE) testing in case-only cohorts	Whether appropriate or not remains to be established	
Estimating the correct genetic model	Visual inspection of Kaplan-Meier curves for the codominant genetic model may reveal the best suitable genetic model for investigation of each polymorphism in multivariable models	Provides a logical and comprehensive solution while also reduces the number of tests to be performed
Minor allele frequency (MAF) of genetic polymorphisms	Excluding the rare polymorphisms (for example, MAF <5%) from the analysis is a common practice	Prevents unstable model construction and by reducing the multiple testing burden and increasing the events/variables ratio also improves the study power
Population stratification due to variable frequencies of genetic polymorphisms in different ethnicities	Detecting and controlling for the population substructure in the cohort eliminates this problem (for example, outlier samples may be eliminated from the analysis or ethnicity can be used as a covariate in the analysis)	Prevents biased estimations and increases the study power
Multiple testing issue due to the investigation of large numbers of polymorphisms	Correction for multiple testing using a variety of methods such as Bonferroni or false discovery rate (FDR) methods [42]	Reduces the false-positive rate (however, ironically may also increase the false-negative rate)
Use of genomic material extracted from archived specimen	Use of new technologies with high rates of successful genotyping [48,49]	Reduces bias and increases study power by allowing the construction of models with a higher number of patients
Use of tumor versus non-tumor DNA in the same study	Using one type (either tumor or non-tumor) depending on the objectives of the study in the cohort or checking the correlation of genotype data obtained from both tumor and non-tumor DNA samples in a set of patients to see whether they are comparable with each other (for example, the tumor DNA may not be a good surrogate for non-tumor DNA all the time)	Prevents bias in study results created by alterations in tumor tissue DNA (that is, different genotypes in tumor DNA compared to non-tumor DNA)

The main characteristics of genetic polymorphisms that require additional considerations in genetic prognostic research are summarized. The majority of the solutions are already applied in susceptibility studies, which can be or have been extended to the prognostic studies.

#### Linkage disequilibrium among genetic polymorphisms provides unique advantages in genetic prognostic research

**Figure 1:**
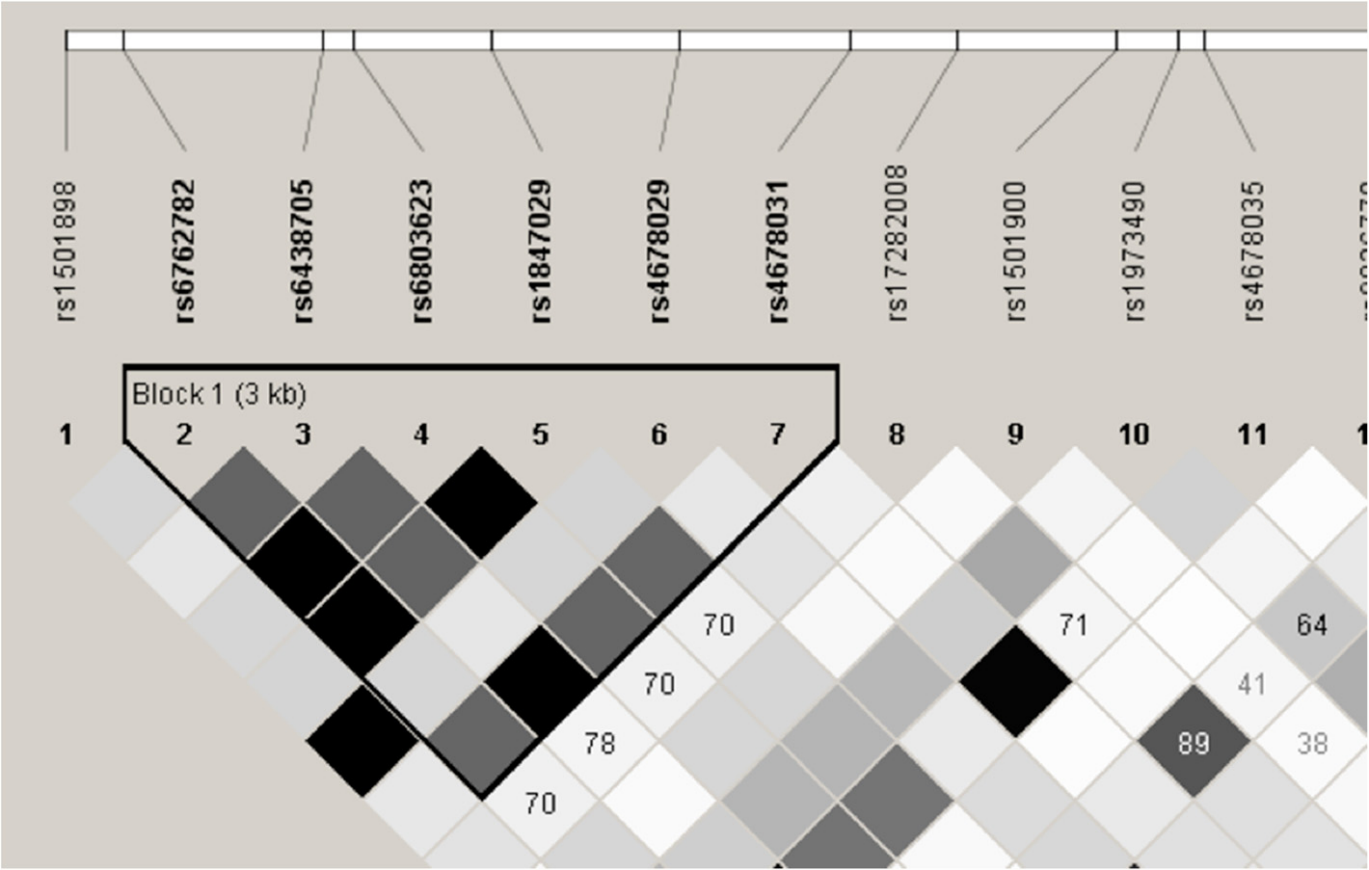
**A partial linkage disequilibrium (LD) map of the human *CASR *(calcium-sensing receptor) gene.** Rs numbers for polymorphisms in this gene are shown at the top. The triangle points to the predicted LD block. The rectangles indicate the correlation coefficient (r^2^) values between the different polymorphisms; the darker the color, the higher the r^2^ values. This figure was obtained using Haploview [22] with the genotype data for Caucasian samples posted at the HapMap database [20,21].

The results of the HapMap project indicated that parts of the human genome are inherited as blocks (called linkage disequilibrium (LD) blocks); the polymorphisms located within these LD blocks are also inherited together with higher probability [20] (Figure 1). Usually, the genotypes of genetic variations in a LD block are also correlated with each other. In prognostic studies, these highly correlated genetic variations create a redundancy problem if investigated at the same time, which may distort the results of the statistical analysis. In order to prevent this problem, a practical alternative is to select a subset of genetic variation that captures the genetic information of correlated variations. This approach involves ‘tagging SNPs (tagSNPs) ’, which can be identified for particular genomic regions for example, through the HapMap website [21] or by using specific software, such as Haploview [22].

In addition to reducing the redundancy among the study variables, analysis of tagSNPs alone may simplify the genotyping efforts and reduce the cost and resources required for genetic prognostic studies [23]. This approach also (by reducing the number of genetic variations to be analyzed) reduces the correction for multiple testing burden (see section entitled The multiple testing issues). Due to these advantages, tagSNPs are increasingly used in genetic prognostic studies [24].

The LD among genetic polymorphisms in close proximity to each other also offers an additional advantage. For instance, once an association between an outcome and a polymorphism is detected and validated, this polymorphism may be considered as a prognostic marker. However, such a genetic marker may not be the direct biological modifier of the prognosis, but rather act as a proxy or surrogate for a nearby ‘true’ prognostic factor (that biologically modifies the risk of outcome). Therefore, after this initial step, the genomic region around the validated polymorphisms should be investigated in detail to identify this prognostic factor. This critical information may then help elucidate the biological basis of variable prognosis in patients, but is unfortunately currently missing in the majority of the genetic prognostic studies. Therefore, future studies may also focus on this missing part of the genetic prognostic research.

#### Genetic polymorphisms may be confounded by other genetic polymorphisms

A confounder is a variable that is linked to both the variable and the outcome [6]. Confounding is a common issue in epidemiological studies and complicates the interpretation of statistical analyses and identification of independent prognostic factors. For example, an association between a variable and an outcome in univariate analyses may be detected due to a variety of reasons, including chance, bias in study design, true association of the variable with the outcome, or the effect of a confounding factor on the variable. Luckily, the multivariate analyses can control confounding by means of adjusting if the confounder and confounded variables are included into the models. However, it is expected that many of the confounder variables are not yet identified, collected for studies, or included into the models in many diseases. Thus, in the absence of their inclusion into the models, it is likely that those variables identified as independent prognostic markers in multivariate analyses remain as confounded by other (unknown or unmeasured) factors.

In the case of genetic variations, it is possible that another genetic variation in the same LD block (for example, a highly correlated or linked polymorphism) may be a confounder. Therefore, in contrast to many other epidemiological confounders, which are difficult to identify, at least some of the genetic confounders may be identified by examining the genomic region of interest and the other genetic variations located in it. Therefore, analysis of genetic variations as prognostic markers also offers unique benefits that do not currently exist in epidemiological research.

#### Should the Hardy-Weinberg equilibrium test be applied in genetic prognostic research?

In genetic research, as a quality control measure, the patient genotypes obtained for genetic polymorphisms are generally checked for genotyping or sampling errors using the Hardy-Weinberg equilibrium (HWE) [25] prior to inclusion into analysis.

HWE states that in an (infinitely) large population with random mating and absence of mutation, migration, or immigration, the allele and genotype frequencies of autosomal loci remain constant over time and follow specific genotype distributions. Therefore, any deviation of genotype distribution from HWE may indicate a population in flux, such as non-random mating and immigration. In genetic research, however, such deviations may also be caused by random fluctuations in samples included into the study, bias in samples collected or included into the research project, presence of samples from different ethnicities (where the frequency of the alleles differ from each other, which is called as population stratification; see section entitled Population substructure of the patient cohort investigated), and technical errors in genotyping (such as underdetection of an allele due to poor primer binding, errors in DNA sampling, DNA contamination by other sources, and errors in interpretation of the genotype [26,27]). In genetic research, HWE has been mostly used to address handling, sampling, and genotyping errors and thus many experts use deviations from HWE as a flag to indicate that the genotyping method may need additional scrutiny.

The conventional approach, for example, in case-control studies is to check for such genotyping errors by applying the HWE test to genotype information in the healthy control population. If the test results do not indicate a deviation from the HWE, then the investigation of this polymorphism in the case-control cohorts proceeds forward. Usually, however, the polymorphisms whose genotype data do not satisfy the HWE are excluded from the analysis. Since, the deviations from HWE may also be due to the other factors (mentioned above), using this test to exclude some genotype data is a conservative approach. In addition, in contrast to the case-control association studies, prognostic research relies only on cases. In our opinion, how appropriate the HWE test in case-only design therefore needs to be debated.

#### Recessive, dominant, codominant, and additive inheritance models: which one to investigate?

Genetic polymorphisms commonly consist of two different alleles (A and B). Thus, a person can carry two copies of the same allele (for example, AA and BB homozygous genotypes) or one of each allele (for example, AB heterozygous genotype). While tri-allelic (for example, AA, AB, BB, AC, and CC genotypes) and quadri-allelic polymorphisms (AA, AB, BB, AC, CC, AD, and DD genotypes) also exist in the human genome, albeit at a much lower frequency, in this article we develop our discussion around the bi-allelic polymorphisms.

Usually, the genetic effects of each of the three genotypes (AA, AB, and BB) on prognosis are unknown and thus consideration of different genetic inheritance models may be required during statistical analysis. Most commonly used genetic models are the recessive, dominant, codominant and additive genetic models [28]. In these genetic models, when the risk allele is unknown (whether it is the major or the minor allele, for example), the genetic model can be defined after the minor allele assuming that the minor allele is the risk allele. In such a case, in the recessive model, patients with homozygous (two) minor alleles (for example, patients with the BB genotype) are considered to be different from the group of patients with both homozygous major allele (patients with the AA genotype) and heterozygote genotype (patients with the AB genotype) in terms of their clinical and prognostic characteristics. In the dominant model, the assumption is that only one minor allele is enough to predict the outcome, thus the patients with BB and AB (containing at least one minor allele) are grouped together and compared with the AA patients. In the codominant model (also called discrete model), the assumption is that the heterozygotes (AB) have a distinct effect, which is different than the effects of minor (BB) and major (AA) homozygote genotypes; therefore, a comparison of these three groups is performed (usually where the major allele homozygotes (AA) serve as a reference group and compared with AB and BB genotypes separately). In the additive model, it is assumed that the effect of heterozygote genotype (AB) is in between the effects of minor homozygote genotype (BB) and major homozygote genotype (AA) in a dose-dependent manner.

Typically, there is no, little, or conflicting biological or phenotype data to hint the right direction to use one inheritance model over another. Therefore, multiple models may need to be investigated in genetic prognostic studies. In our experience, the most common and robust genetic models investigated are the additive and the codominant models. In order to completely evaluate the role of a polymorphism with prognostic characteristics of patients, however, ideally all possible genetic models require investigation. Ironically, this also increases the number of statistical tests performed, which may require correction for multiple testing (see section entitled The multiple testing issues). In addition, the correction for multiple testing procedures almost always reduce the statistical power. Therefore, as a way of overcoming the multiple testing issue, many researchers opt for application of only one or a few of these inheritance models in their studies, rather than investigating multiple models for a comprehensive analysis. Such a practice, however, may lead to omission of potentially important findings.

In addition to the correction for multiple testing, analysis of multiple genetic models in the same study presents another challenge. In univariate analysis, detection of a significant association of a polymorphism with an outcome in more than one genetic model is not uncommon. Especially if the models contain multiple polymorphisms, constructing a separate multivariate model for each inheritance model, for example, is not a logical solution as each polymorphism may affect the prognosis under different inheritance patterns.

**Figure 2:**
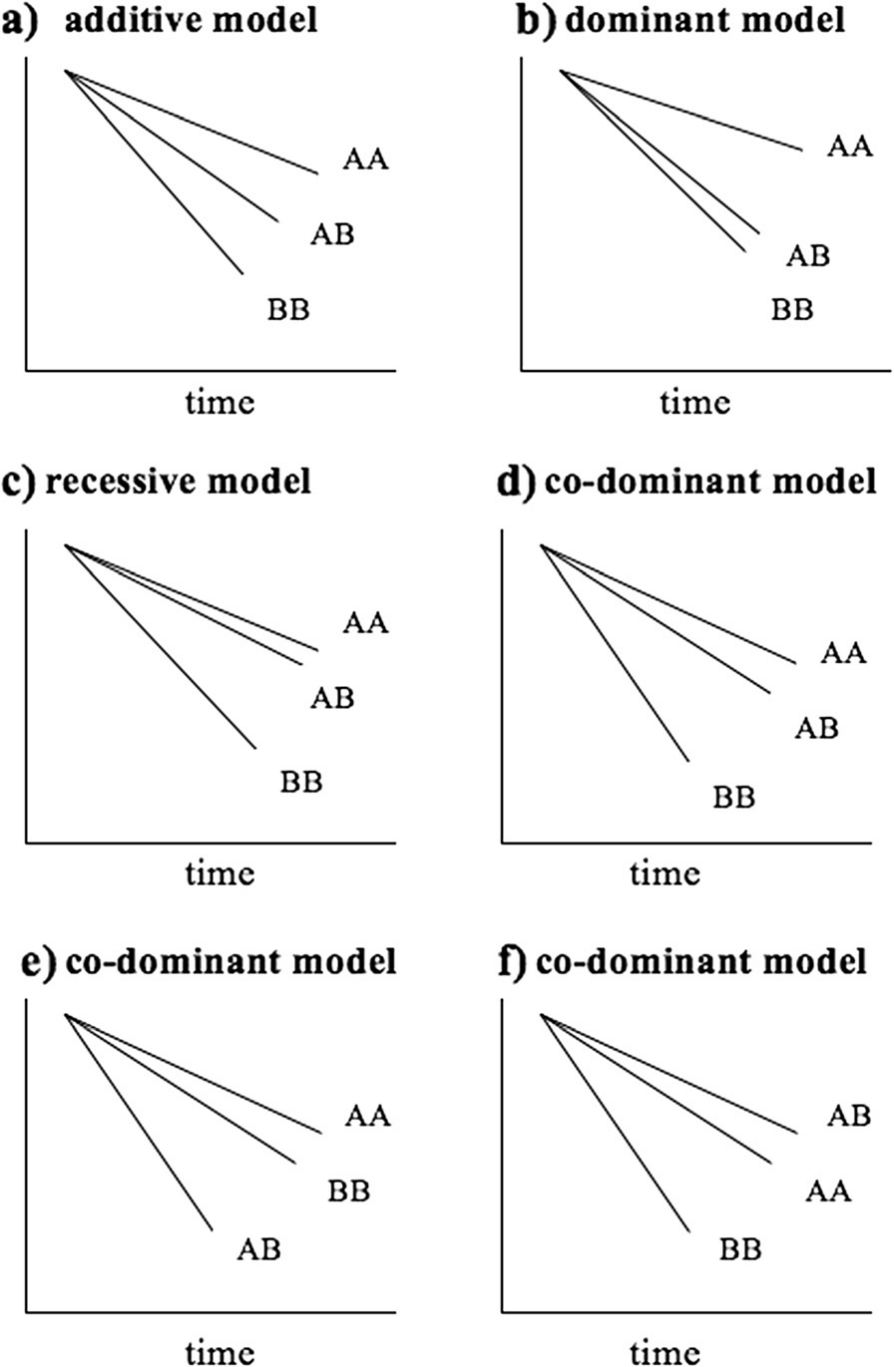
**Kaplan-Meier curves may identify the best fitting genetic model for a polymorphism.** For simplicity, survival curves are shown as straight lines. AA = major allele homozygous genotype, AB = heterozygous genotype, BB = minor allele homozygous genotype, assuming allele ‘A’ is the common allele. **(a)** The effect of the AB genotype on survival is approximately half between the AA and BB genotypes, thus the additive model is appropriate for this polymorphism in the multivariate analysis. **(b)** The curves of AB and BB genotypes cluster closer to each other when compared to the AA genotype’s curve, thus, the effect of the polymorphism is likely to be dominant. **(c)** AA and AB genotype survival curves cluster together and clearly separate from the BB genotype curve. Thus, the inheritance pattern is likely to be recessive. **(d)** In this case, the effect of AB genotype is somewhat in between the effects of AA and BB genotypes, thus, analyzing this polymorphism assuming the codominant model is suitable. **(e)** This is an interesting polymorphism where the heterozygotes are associated with worse survival compared to either homozygous genotypes (AA and BB). The codominant genetic model is the appropriate model to investigate such polymorphisms in multivariate analyses. Exact biological and genetic reasons for such associations are not clear, but it may be due to heterozygote disadvantage where the heterozygotes display phenotype but not the either homozygotes. **(f)** The heterozygotes have better survival than AA and BB homozygotes. This case may represent a ‘heterozygote advantage’ situation, where the heterozygotes have favorable survival characteristics. Similar examples are observed in Mendelian diseases, such as sickle cell anemia [56]. In both (e) and (f), presence of another genetic variation in close proximity acting as a prognostic factor (which is not highly correlated with this polymorphism) may be an alternative explanation.

One solution to this issue is to determine the best pattern of genetic effect (that is, inheritance model) for each polymorphism by inspecting the univariate Kaplan-Meier survival curves conducted for the codominant genetic model (Figure 2). This way, the genetic model for each polymorphism may be determined prior to performing multivariate analysis, which circumvents the concern of multiple and blind looks at the data and unnecessary multiple modeling. Another solution is to test the association of a genetic variation with prognosis under multiple genetic models in separate univariate analyses and then to select the best genetic model among all based on the *P* values (that is, the lower the *P* value, more appropriate is the genetic model to detect the effect of the variation) [29].

#### The minor allele frequency of polymorphisms and the other determinants of the study power in the multivariable models

Genetic polymorphisms present in a range of minor allele frequencies (MAFs) in human (1% to 50%). The minor allele frequency of a polymorphism is critical information that helps determine the inclusion of the polymorphism into the statistical analysis, as rare variables may hamper the model construction [6]. For example, a polymorphism with a MAF of 1% studied in a cohort of 1000 chromosomes (that is, 500 patients, assuming it is on an autosomal chromosome) will be detected in only 10 of the chromosomes. Therefore, the study analyzing this polymorphism (in univariate or multivariate analyses) may not have enough power (that is, the probability of detecting a significant association when it actually exists). Therefore, as a general rule, as the MAF of a polymorphism increases, so does the study power. Therefore, exclusion of polymorphisms with a MAF of <1% or <5% is a common practice in current genetic prognostic studies. However, exclusion of rare genetic variations may also lead to missing the identification of rare variations that have strong effects (for example, high HRs) on prognosis. Study power is also directly related to the size of the effect that a polymorphism has on the outcome; to detect smaller effects, larger sample sizes are required [30], yet to detect prognostic markers (whether rare or common) with strong effects, studying a cohort with a relatively smaller size may be sufficient [4].

Finally, in addition to the sample size and the effect size, we should also mention that the number of events per investigated variable in a multivariable model may impact the study power. A recommended rule for statistical power in multivariate analyses is the presence of at least ten events per independent variable [6]. The event/variable ratio is usually high in cohorts with high risk of events (such as metastatic cancer patients) or in cohorts with long follow-up periods that allow detection of most events experienced by the patients. Thus, in the genetic prognostic research, when an association is not detected, the role of insufficient study power as well as inadequate follow-up time should be considered before concluding that the polymorphism is not an independent predictor of prognosis. This event per variable ratio also demonstrates the need of entering a relatively small number of variables into the multivariable models.

#### Population substructure of the patient cohort investigated

Most of the genetic prognostic studies are based on population-based design with unrelated patients. However, these studies are prone to population stratification [31]. Population stratification refers to different allele frequencies of subpopulations in the study cohort due to an ancestry difference in study patients (for example, patients from different ethnicities). The influence of stratification on genetic association studies has been demonstrated even in well-designed protocols, with greatest effect in admixed populations (such as African-Americans) and for diseases with different variant prevalence rates in the ancestral populations [32]. For example, allelic frequencies of certain polymorphisms may significantly differ among Caucasians, Asians, and Africans [33,34]. An example to such a polymorphism is the (TA)7 allele in the *UGT1A1* gene (responsible for the detoxification of the active metabolite of the chemotherapeutic agent, irinotecan), which is more common in Caucasians than in Asians [35]. In addition, allelic frequencies of polymorphisms may also differ within each of these populations (such as among different populations from countries in Europe) [36]. Therefore, unrecognized population stratification can lead to biased estimation (for example, inflated false-positive results), or reduce statistical power if not appropriately corrected [37].

Since many cohorts investigated contain patients from different ethnicities and with potential population substructure, various methods have been developed to detect and control for population stratification in human genetic association studies, which may also be applied to genetic prognostic research; (a) the genomic control method [38] corrects for population stratification by adjusting with a variable determined from a set of random genetic markers that are not associated with the disease outcomes in the studied cohort, (b) structured association can assign the study patients to distinct subpopulations and then aggregates evidence of association within each subpopulation. The most commonly used genetic package for structured association analysis is the STRUCTURE program [39], (c) a recent development for the correction of population stratification utilizes EIGENSTRAT [40], which computes principal components for collected SNPs (for example, across the genome in genome wide studies) to identify population structure. In this approach, the top principal components that contribute mostly to the genetic variation in the study cohort are included as covariates in multivariate regression models to adjust for population stratification. Using these or other similar methods to identify and account for the population stratification in genetic prognostic research may, therefore, improve reliability of results.

#### The multiple testing issues

When multiple hypotheses are tested in a study, using the significance level at the traditional value of 0.05 may lead to inflated false-positive results. In other words, the more comparisons we perform, the more likely we can obtain a significant result by chance. While for candidate gene studies, a modified significance threshold (for example, *P* <0.0005; [41]) was suggested, with the assistance of high-throughput genotyping technologies, genetic prognostic studies are increasingly investigating larger numbers of polymorphisms (for example, genomewide scans). This increase in the number of polymorphisms creates a challenge of how to deal with the multiple testing issue.

A variety of statistical correction methods have been developed [42] and may be applied to genetic prognostic research investigating large numbers of genetic markers. The most commonly used methods for multiple-testing correction are Bonferroni adjustment, permutation algorithm, and the false discovery rate (FDR) methodology. The Bonferroni adjustment is useful when the number of multiple testing is not very large and the tests are independent (for example, candidate gene study with genetic variants that are not in LD with each other). However, Bonferroni adjustment may be too conservative when the tests are not independent, which is often the case in genetic prognostic studies where the polymorphisms to be tested are in high LD. Nyholt [43] has proposed a correction method based on estimation of the effective number of independent tests. Permutation-based adjusted significance levels are particularly useful when there are strong dependencies among the tests. However, the computation is quite intensive. FDR methodology is suitable for very large scale multiple testing [44]. The statistical significance thresholds can be set according to the overall pattern of results [45]. To improve power, the FDR method can be weighted according to the importance of the test [46] such as evidence from linkage scans [47]. While Bonferroni adjustment can be performed manually, specific statistical programs are required for both permutation-based and FDR adjustments.

While the correction for multiple testing procedures aim to reduce the false-positive findings, there are other ways to help limit the number of spurious findings. For example, although not completely ideal, internal validation techniques such as cross-validation and bootstrap may be applied to the cohort data to reduce the false-positive discoveries [6,11,12]. The best approach to test whether a positive association is a true association, however, is to replicate the findings in another patient cohort preferably from another center or population [11].

#### Use of genomic material extracted from achieved specimen may require extra care in the genetic prognostic studies

The majority of prognostic studies have been conducted on retrospective cohorts collected for other purposes. Here the availability of genetic material and its efficiency in yielding the genotypes are not usually considered optimal. For example, in a significant portion of studies in cancer, formalin-fixed-paraffin-embedded (FFPE) tissue blocks (from both tumor and adjacent non-tumor tissue) collected during surgery are used to extract genomic DNA. The quality and the amount of this type of DNA may not be high and is susceptible to lower genotyping yields. This limitation in quantity and quality of the genomic DNA in retrospective cohorts usually restricts the options on suitable genotyping technology and the extent of the genotyping analysis (for example, limiting the number of genes/polymorphisms that can be investigated). An increased proportion of failed genotyping may also create biased study results. Recently a few studies have shown that the limitations of FFPE-extracted DNA can be overcome by certain genotyping methods [48,49]. In addition, recent prognostic studies have been keen about banking blood samples that contain DNA (that is, whole blood or leukocytes). These technological and study-design-related advances are expected to improve the genotyping success rates and reduce bias, and thus increase the capacity and reliability of the future genetic prognostic research.

#### Use of tumor DNA versus non-tumor DNA in genetic prognostic research: which one is appropriate?

Due to the availability of the tumor tissue in many studies and the fact that disease progression and prognosis of cancer patients are affected by tumor behavior [50-54], analysis of the tumor genotypes may be feasible and can yield interesting and valuable prognostic information. However, distinct differences between the tumor and non-tumor extracted DNA samples of a single patient create a challenge. For example, frequent, somatic small-scale (such as point mutations) and large-scale (such as aneuploidy and loss-of-heterozygosity (LOH)) alterations are well-known characteristics of the cancer genomes. Therefore, tumor DNA and non-tumor DNA of the same individual may have different genotypes for a given polymorphism.

In prognostic research in oncology, the optimal source of DNA depends on the study aims. If the association being tested is toxicity, then the optimal DNA may be the DNA in the target organ of the toxicity (for example, skin for rash, gut for diarrhea) or the organ that metabolizes, excretes, or activates the active drug (for example, liver, kidney, biliary track). Surrogate DNA in this case may come from any germline (that is, non-tumor) source, such as blood. In addition, if the mechanism influences the host stroma (that is, angiogenesis), the optimal source of DNA is from the host tissue (that is, non-tumor surgical tissue). In contrast, if the association relates to efficacy and the polymorphism influences the tumor itself (for example, by affecting the proliferative capacity or oncogenic pathways in tumors), then the most appropriate source of DNA is the tumor itself. Since it is impractical to obtain multiple sources of DNA to test different hypotheses in the same patient population, one can test tumor and non-tumor DNA for their correlation in genotype. High concordances (that is, above 95%) may suggest that the polymorphism itself is not a hotbed of somatic change in the tumor and thus allow tumor and non-tumor tissue to become surrogates for each other.

## Conclusions

Genetic prognostic research examining the relation and predictive value of genetic polymorphisms is a promising and rapidly developing research area. In contrast to other variables commonly studied, genetic polymorphisms have several unique characteristics that require special considerations in study design and data analysis. While some of these characteristics (such as linkage disequilibrium among genetic polymorphisms and tagSNPs) ease our efforts, other characteristics (such as different frequencies of polymorphisms in different ethnicities and use of genomic material extracted from archived specimen) may bias our results, if left unaccounted for. In addition, variability in study design and analysis can adversely affect advancement of the genetic prognostic research and translation of its results into the clinic. Recommendations modeled as guidelines (for example, REMARK guidelines [55]) on how to conduct and compare genetic prognostic research involving genetic markers may be needed to expedite this exiting and promising research field.

## Abbreviations

CNV: copy number variation; FDR: false discovery rate; FFPE: formalin-fixed paraffin-embedded; HWE: Hardy-Weinberg equilibrium; HR: hazard ratio; indel: insertion and deletion; LD: linkage disequilibrium; LOH: loss-of-heterozygosity; MAF: minor allele frequency; OR: odds ratio; SNP: single nucleotide polymorphism; tagSNP: tagging SNP

## Competing interests

The authors declare that they have no conflicts of interest related to this work.

## Authors’ contributions

SS initiated the idea, contributed to the design and organization of the manuscript, drafted and submitted the manuscript. GL contributed to the conception, organization and design of the manuscript, drafted and critically reviewed the manuscript. WX contributed to the conception, organization and design of the manuscript, drafted and critically reviewed the manuscript. All authors read and approved the final manuscript.

## Pre-publication history

The pre-publication history for this paper can be accessed here:

http://www.biomedcentral.com/1741-7015/11/149/prepub
